# A hyper-dynamic nature of bivalent promoter states underlies coordinated developmental gene expression modules

**DOI:** 10.1186/1471-2164-15-1186

**Published:** 2014-12-30

**Authors:** Akshay Shah, Anja Oldenburg, Philippe Collas

**Affiliations:** Department of Molecular Medicine, Institute of Basic Medical Sciences, Faculty of Medicine, University of Oslo, 0317 Oslo, Norway; Norwegian Center for Stem Cell Research, Oslo University Hospital, Oslo, Norway

**Keywords:** Chromatin state, Differentiation, Hidden Markov Model, Promoter, Transcriptome

## Abstract

**Background:**

Chromatin remodeling is crucial for proper programing of developmental gene expression. Recent work provides a dynamic view of post-translational histone modifications during differentiation; however there is little insight on the evolution of combinatorial genome-wide patterns of chromatin marks, excluding an essential aspect of developmental gene regulation.

**Results:**

We report here a 15-chromatin state Hidden Markov Model which describes changes in chromatin signatures in relation to transcription profiles during differentiation of human pre-adipocytes into adipocytes. We identify nineteen modules of gene expression reflecting multiple waves of transcriptional up- and down-regulation which characterize adipogenic differentiation. From our model, we developed chromatin state matrices fitting each of these transcription modules to show how the complexity and dynamic nature of chromatin signatures relate to expression patterns. Spatial relationships between chromatin states underlie a high-order chromatin organization in differentiating adipocytes. We show the importance of gene expression level in generating diversity in chromatin signatures, and show that the hyper-dynamic nature of H3K4me2/H3K27me3-marked ‘bivalent’ promoter states underlies many of the gene expression patterns associated with adipogenic differentiation.

**Conclusions:**

Our results reveal the highly dynamic nature of bivalent promoter states within the adipogenic lineage. The data constitute a valuable resource enabling the assessment of possibilities to alter the adipogenic program.

**Electronic supplementary material:**

The online version of this article (doi:10.1186/1471-2164-15-1186) contains supplementary material, which is available to authorized users.

## Background

Developmental gene expression entails waves of coordinated transcriptional activation and repression events, which are orchestrated at least in part by the epigenome [1–5], a layer of reversible post-translational modifications on chromatin [6]. These studies invariably indicate that the epigenome is dynamic and that epigenetic modifications are linked to changes in gene expression in a concordant or sometimes seemingly non-concordant manner. However, some features of the epigenome appear to be more static than others: subsets of histone post-translational modifications (hPTMs) form combinatorial associations that are relatively stable and hence can be used to functionally annotate genomic elements [7, 8].

Several stem or progenitor cell-based differentiation models have been used to assess the temporal changes in epigenetic states across the genome [9–11]. A system which has remained under-studied relative to its societal importance is the differentiation of pre-adipocytes into adipocytes. Regulation of the balance between maintenance of a pool of adipocyte progenitors and their differentiation into adipocytes is essential for adipose tissue homeostasis [12, 13]. Adipogenic differentiation is driven by activation of genes encoding transcription factors that synergistically up-regulate target genes involved in adipocyte formation and lipid metabolism [14–16], and by chromatin remodeling notably at regulatory elements essential for transcription factor binding [17]. Genome-wide profiling of hPTMs by chromatin immunoprecipitation and high-throughput sequencing (ChIP-seq) during mouse and human *in vitro* adipogenic differentiation has led to the identification of thousands of putative adipogenic-specific promoters and enhancers [18]. The current data portray a dynamic view of enrichment in individual hPTMs [17, 18]; however insights on the temporal transitions in combinatorial associations of hPTMs during adipogenic differentiation have been missing, leaving out a key aspect of developmental gene regulation.

A deeper appreciation of the complexity of chromatin signatures can be obtained through Bayesian methods which model combinatorial associations of chromatin marks [19, 20]. Among these, Hidden Markov Modeling (HMM) uses machine learning to discover chromatin states from recurrent combinations of histone modifications, transcription factors and chromatin remodeling factors [19, 21]. From the analysis of a panel of factors in several unrelated cell types [1, 21–25], HMM provides the ability to distinguish functional genomic elements, generating genome-wide profiles of chromatin ‘activity’ [19, 21]. However, the experimental material used in these studies was not chosen to infer a temporal dynamics of chromatin states; thus developmental transitions in chromatin states in a genome-wide context have not been fully explored.

To palliate this gap, a variation of HMM has been proposed as an unsupervised hierarchical model enabling correlations between gene expression patterns and clusters of combinatorial chromatin marks [9]. This model supports the tissue- and cell type-specificity of enhancer activity and associated histone modifications [10, 21, 26], and concurs with recent evidence for distinct mechanisms of gene expression regulation along the genome [27].

Here, we applied ChromHMM, a high-throughput pipeline based on a multivariate HMM [19], using ChIP-seq data for seven chromatin marks [18], in combination with RNA-sequencing (RNA-seq), to discover interrelationships between chromatin states and gene expression patterns during differentiation of human pre-adipocytes. We identify several coordinated gene expression modules and learned a 15-state model which we use to map and quantify temporal transitions in chromatin states across these expression modules. We reveal the importance of gene expression level in generating diversity in chromatin signatures and the hyper-dynamic nature of ‘bivalent’ promoter states during lineage-specific differentiation.

## Results

### Coordinated gene expression modules characterize adipogenic differentiation

**Figure 1:**
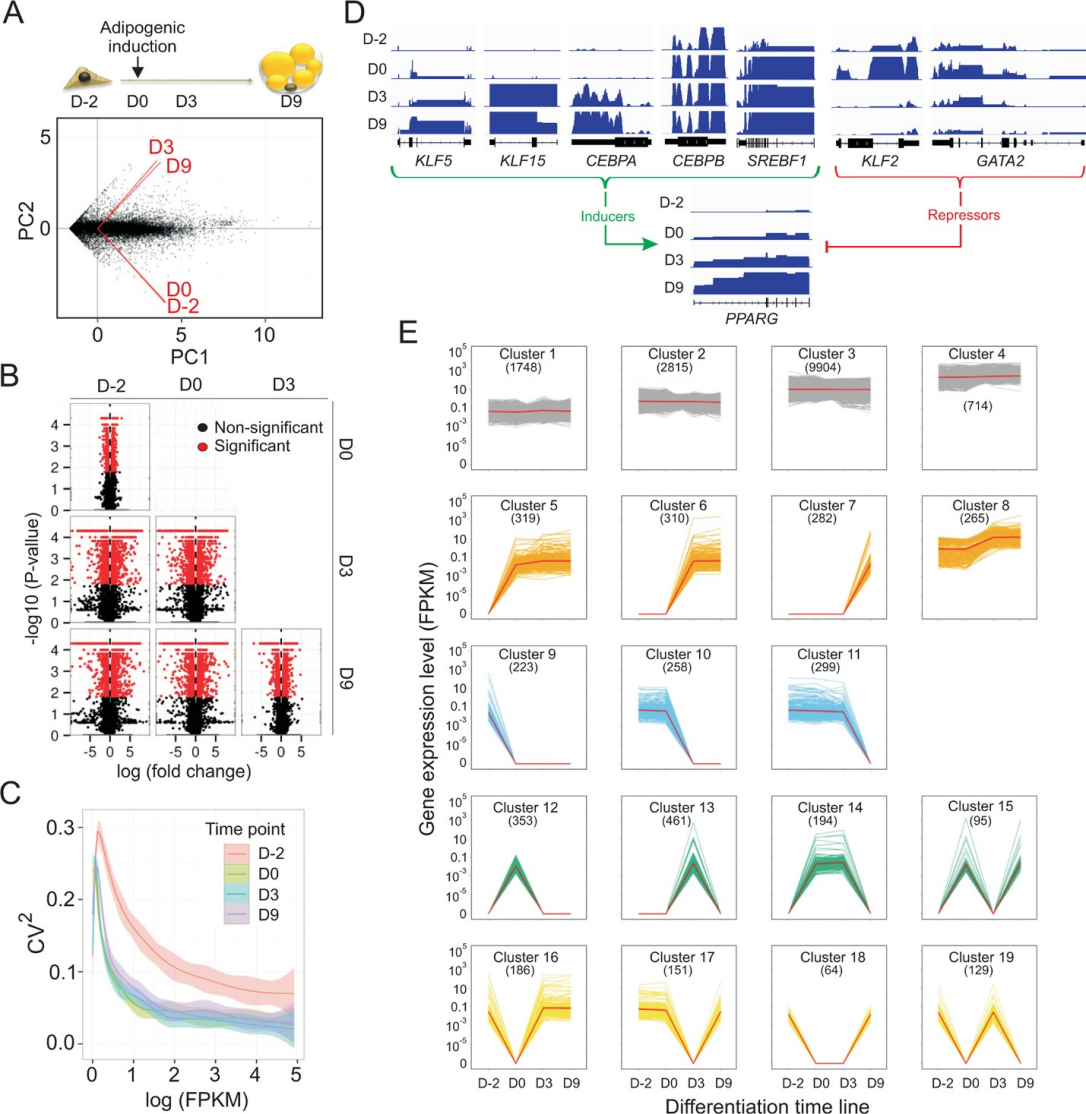
**Coordinated gene expression modules during adipogenic differentiation. (A)** Principal component analysis of differentially expressed genes. **(B)** Fold change in expression level of each gene plotted against its P-value (expressed in -log10 FPKM). Significance: fold change > 2; α < 0.05. **(C)** Square coefficient of variance (CV^2^) of expression as a function of expression level (log FPKM) at each differentiation time point. **(D)** RNA-seq profiles of inducers and repressors of *PPARG* expression. Scales (FPKM): *KLF15*, 0-25; *KLF5*, 0-10; *CEBPA*, *CEBPB*, *SREBF1*, *KLF2*: 0-50, *GATA2*: 0-50, *PPARG*, 0-250. **(E)** Hierarchical clustering of genes into 19 expression modules throughout differentiation. Each line represents one transcript. Numbers of genes in each cluster are shown. FPKM scales are constant for each cluster.

We used deep paired-end RNA-seq to unveil transcriptomic changes at four time points of adipogenic differentiation of human preadipocytes. Numbers of reads per time point ranged from 40 to 102 million, of which numbers of paired alignments were >30 to > 95 million (Additional file 1: Table S1). We analyzed cells two days before adipogenic induction (D-2; undifferentiated proliferating ASCs), immediately prior to adipogenic induction (D0; undifferentiated confluent cells, 48 h after growth factor removal) and on D3 and D9 of differentiation. Principal component analysis shows that the first two components segregate the transcriptome of proliferating and confluent ASCs from that of adipogenic-stimulated cells (Figure 1A), reflecting a major transcriptional switch. This conversion is manifested by the greatest differences both in the numbers of differentially expressed genes (2512 and 2910 up- and down-regulated genes respectively; Additional file 1: Figure S1A) and in the magnitude of differential expression levels (Figure 1B; see Additional file 2: Table S2 for lists of differentially expressed genes). Culturing ASCs to confluency remarkably reduces the overall variability in transcript levels detected in proliferating cells (Figure 1C), consistent with greater individual variations in gene expression patterns in unsynchronized cell populations [28]. This reduced variability is maintained after adipogenic induction (Figure 1C), consistent with the establishment of a coordinated gene expression program. Of note, this does not preclude potentially persistent cell-to-cell variations in transcriptional response to the differentiation stimulus in the populations analyzed [29].

The transition from cell proliferation to confluency (D-2 to D0) is characterized by up-regulation of genes involved in extracellular matrix reorganization, regulation of proliferation, development and signaling, and down-regulation of genes important for cytoskeletal reorganization and signaling functions (P < 10^−6^ – 10^−12^; Additional file 2: Table S3), in line with the acquisition of a cell cycle arrest phenotype. Adipogenic stimulation (D0-D3 transition) up-regulates metabolic genes key for adipocyte development including lipid synthesis, metabolism and homeostasis (P < 10^−8^ – 10^−28^; Additional file 2: Tables S2 and S3). We importantly confirm expression and up-regulation of positive regulators of PPARγ (peroxisome proliferator-activated receptor γ), a key regulator of adipogenesis [15] (Figure 1D), including Krüppel-like factors (*KLF4*, *KLF5*, *KLF6*, *KLF9* and *KLF15*), STAT proteins (*STAT5A*, *STAT5B*), sterol-response element binding protein 1c (SREBP1c; *SREBF1*) and CCAAT/enhancer-binding proteins (C/EBPs; *CEBPA*, *CEBPB*, *CEBPD*, *CEBPE*). Conversely, negative regulators of PPARγ (*KLF2* and *GATA2*) are down-regulated (Figure 1D; Additional file 2: Table S2). Our RNA-seq data thus identify massive transcriptional changes leading to the activation of key metabolic genes required for adipocyte formation and function.

To provide a dynamic assessment of transcriptional changes during differentiation, we identified by hierarchical clustering 19 cohorts of genes displaying unique expression profiles throughout differentiation (Figure 1E). We identify cohorts of genes with stable expression levels (clusters 1-4) which regroup 81% of all expressed genes. The remaining genes partition into 15 clusters showing sequential transcriptional induction (that is, up-regulation from zero FPKM) on D0, D3 or D9 (clusters 5-7), up-regulation of already expressed genes (cluster 8), sequential transcriptional down-regulation (to zero FPKM; cluster 9-11), and transient up- or down-regulation (cluster 12-19). Genes sequentially activated or repressed (clusters 5-7, 9-11) or transiently activated or inactivated (clusters 12-19) are involved in signaling and transcription regulation processes important for adipogenic and lipid metabolism functions encoded by cluster 8 (Additional file 1: Figure S1B; Additional file 2: Tables S4 and S5). Our RNA-seq and clustering data reveal therefore the establishment of coherent gene expression modules characterizing the adipogenic differentiation program. Our datasets also constitute a high-depth transcriptome resource mapping the adipogenic process in human primary preadipocytes.

### Spatial relationships between chromatin states reveal restricted state transition choices along the genome

**Figure 2:**
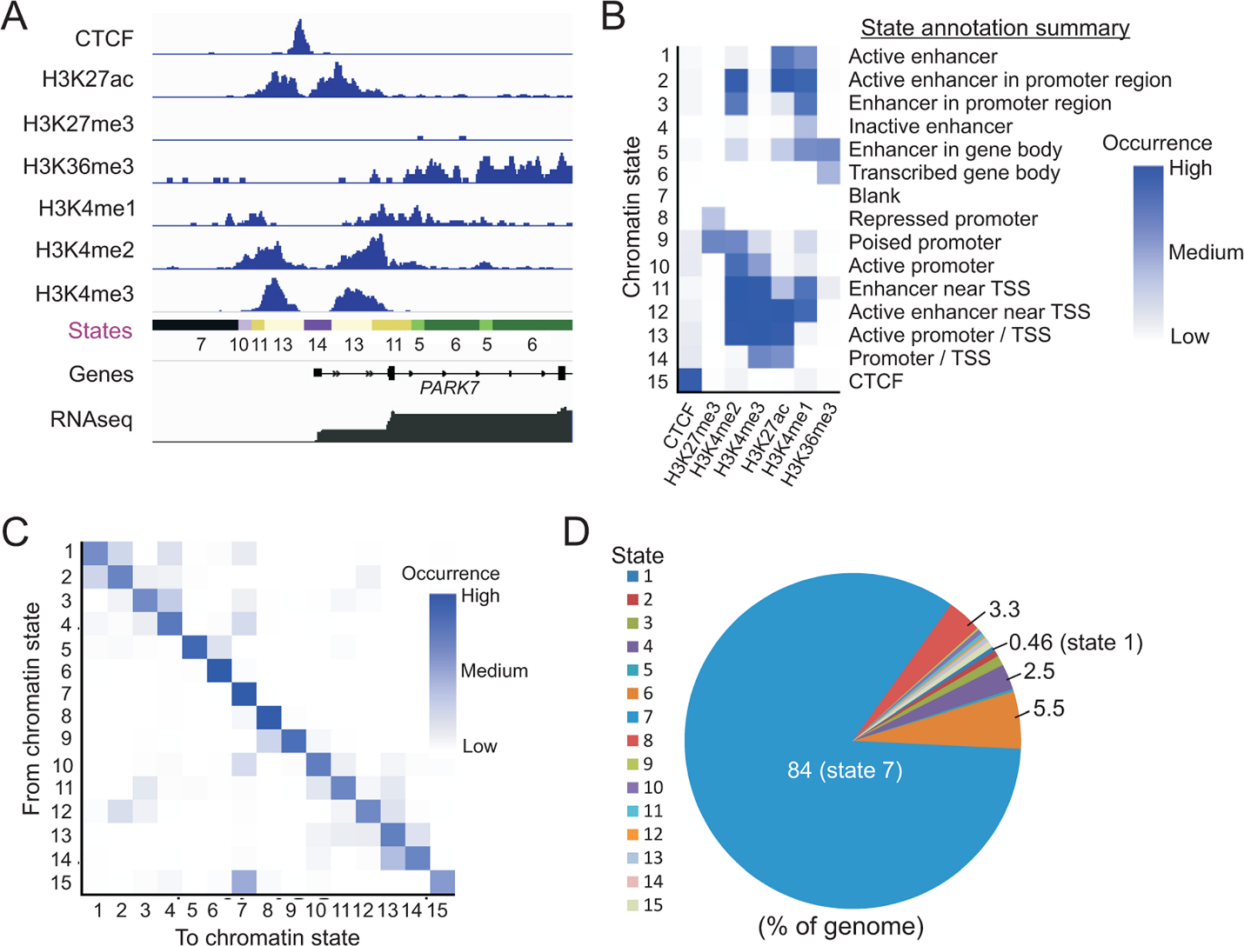
**Chromatin states learned from recurrent combinations of seven chromatin marks in proliferating and adipogenic-stimulated preadipocytes. (A)** Chromatin modifications and resulting chromatin states (color coded) in a 6 kb region of chromosome 1. **(B)** ChromHMM emission parameters and functional element annotation from the 15 states. Scale reflects enrichment of a given state in indicated chromatin marks. **(C)** Transition parameters across the genome. Scale reflects the frequency of occurrence for a give state. **(D)** Proportions of the genome covered by the 15 chromatin states learned in our model.

We sought to identify a relationship between temporal gene expression changes and enrichment in combinations of chromatin marks during adipogenic differentiation. We used ChromHMM [19] to learn a 15 chromatin state (‘cs’) model from recurrent combinations, in consecutive 200-base pair (bp) bins, of seven chromatin marks (H3K27me3, H3K27ac, H3K4me1, H3K4me2, H3K4me3, H3K36me3 and the CCCTC-binding factor CTCF) profiled by ChIP-seq [18] in preadipocytes on D-2, 0, 3 and 9 of differentiation (Figure 2A). Our model balances information level, interpretability and resolution. We annotated the 15 states into functional genomic elements including active and inactive enhancers, enhancer location (near transcription start sites, within promoters or within gene bodies), active, inactive and ‘bivalent’ promoters and transcribed gene bodies (Figure 2B). ChromHMM also generates transition parameters based on spatial relationships between adjacent genomic segments, representing the sequence of states across the genome (Figure 2C). These parameters show that one state is most commonly followed by the same state or by one other state rather than by many states (Figure 2C). For instance, cs1 (H3K27ac/H3K4me1, annotated as active enhancer sites) and cs2 (H3K27ac/H3K4me1/H3K4me2; active enhancers in promoter regions), or cs3 (H3K4me1, H3K4me2) and cs4 (H3K4me1), frequently follow each other, suggesting embedding of active or inactive enhancers within promoter regions. State 4 can also be followed by ‘blank’ state 7, revealing enhancer sites also within chromatin deserts (Figure 2C).

We find that genome coverage and length of states vary considerably, from 200 bp (a bin size) to tens of kilobases (Additional file 1: Figure S2A). This agrees with the genomic nature of histone modifications defining these states, which mark spatially restricted elements (e.g. promoters or enhancers; cs1) or wider areas such as H3K27me3 domains (cs8) or entire H3K36me3-marked coding regions (cs6) (Figure 2A,D). Our model also enables to estimate that ~16% of the genome contains at least one of the marks examined (Figure 2D). This is in line with recent data from man [21], mouse [9] and *Drosophila* [22, 30], which based on the marks examined and analysis methods concur in that most of the genome is in a ‘blank’ state. Logically, state 7 is the broadest (Figure 2D; Additional file 1: Figure S2A) and essentially deprived of genes (4.3×10^−4^ genes per megabase; data not shown). The spatial relationship between chromatin states learned in our HMM demonstrates a limited choice in the the transition from one state to another along the genome, which reflects a higher order chromatin layout. The data also reveal the existence of chromatin state ‘variants’ learned from non-canonical genomic elements, such as canonical enhancer sites (marked by H3K4me1 with or without H3K27ac) within promoter elements or active gene bodies.

### Analyzing the load and temporal dynamics of chromatin states

**Figure 3:**
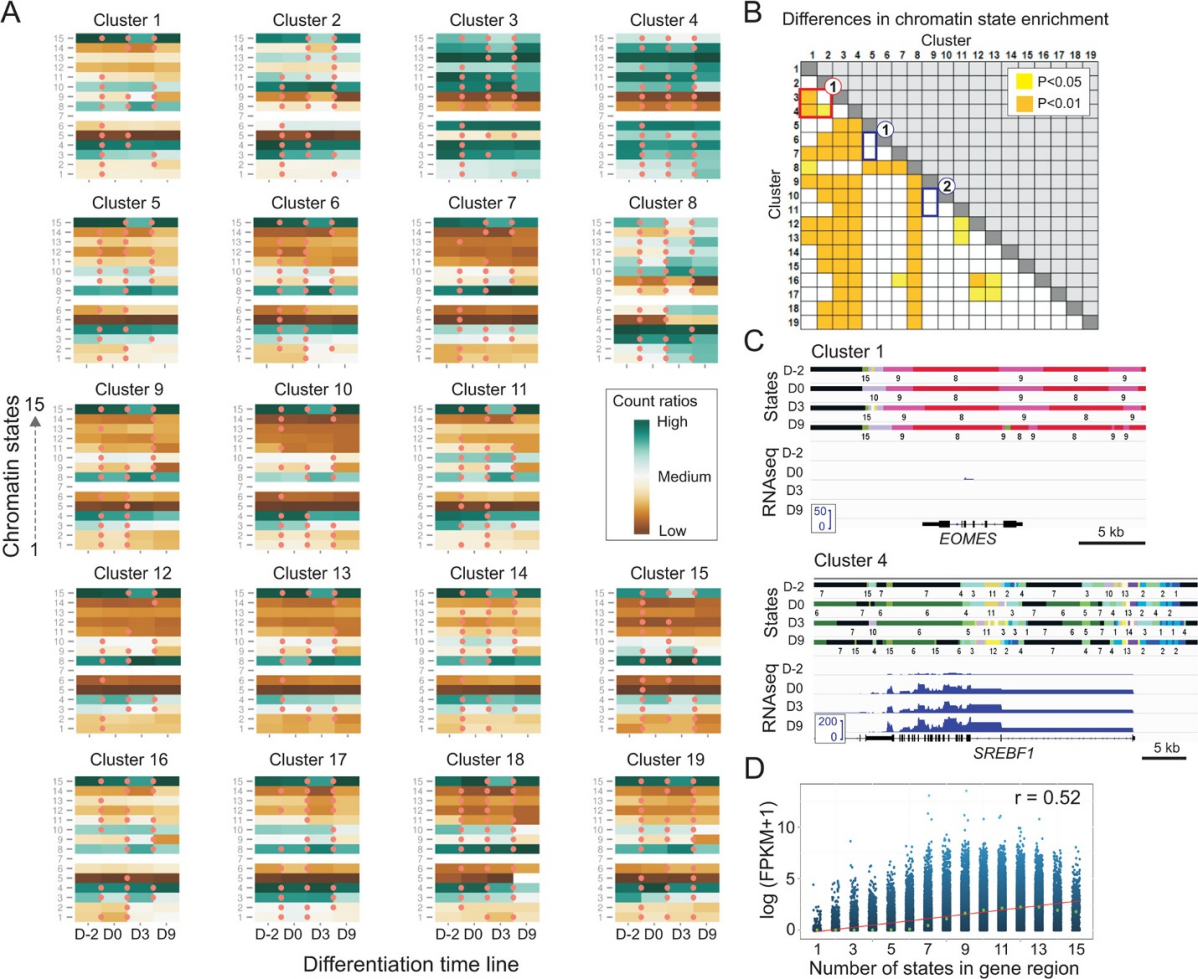
**Gene expression level correlates with chromatin signature complexity. (A)** Heat maps of chromatin state enrichment in gene regions (gene length ± 10 kb) for each expression cluster during adipogenic differentiation. Gene ratios (see text) at each differentiation time point are scaled from low-to-high and reflect chromatin state ‘loads’. Time point transitions at which a difference in gene ratios is significant are marked (dot; P < 0.05; t-test with Bonferroni correction). **(B)** Matrix of statistically significant differences in global state enrichment between expression clusters; P-values are shown in Additional file 2: Table S6A; Wilcoxon test with Bonferroni correction. **(C)** Chromatin states and RNA-seq profiles at the inactive *EOMES* locus and at the expressed adipogenic *SREBF1* locus; FPKM ranges are shown. **(D)** Regression analysis of gene expression level as a function of the number of chromatin states, i.e. complexity of chromatin signatures, in gene regions.

We next established relationships between chromatin state enrichment level or state dynamics in the course of differentiation, and gene expression outputs. We computed, for each expression cluster, the ratios of genes (defined as gene body ± 10 kb) harboring any given state at a given time point. The output was normalized chromatin state heat maps linked to genes at each time point (Figure 3A; see Methods). We also determined time points at which gene ratios significantly differ from the previous or following time point (P < 0.05; t-test with Bonferroni correction; Figure 3A, red dots). Since a difference in gene ratios for any given state between two time points represents a gain or loss of that state for these genes, the maps in effect show the levels of enrichment of genes in a given chromatin state at each time point, and significant changes in chromatin state between time points. Normalization of gene ratios, i.e. chromatin state enrichment levels, within expression clusters allows chromatin state maps to be compared between clusters.

### Strong gene expression is associated with high chromatin signature complexity

We first examined the relationship between gene expression patterns and global chromatin state enrichment level (or ‘load’). From the expression cluster-based chromatin state maps, we computed a statistics matrix of significant differences in global chromatin state enrichment between expression clusters (Figure 3B). To this end, we averaged all gene ratios within each cluster for all states (excluding state 7) and compared ratios between clusters. We find that strongly expressed genes (cluster 3, 4, 8) display greater chromatin state enrichment than weakly expressed genes (clusters 1, 2) (Figures 3A,B; red box 1; P < 0.01, Wilcoxon test; see Additional file 2: Table S6A for P-values). This is exemplified for individual genes: *EOMES*, essentially not expressed in ASCs or adipocytes (cluster 1) is marked by bank state 7 and the repressive states cs8 and cs9, whereas the pro-adipogenic factor *SREBF1*, expressed at all stages (cluster 8), is heavily enriched in states (Figure 3C; see also Additional file 1: Figure S2B). Clusters of strongly expressed genes (clusters 3, 4, 8) also display greater state enrichment than all other clusters (clusters 9-19; Figure 3A,B), which can be related to the lower expression level in the latter (see Figure 1E). Linear regression analysis shows that gene expression level correlates with global enrichment of chromatin in hPTMs (Pearson correlation 0.52; Figure 3D). This would be expected from the nature of the hPTMs examined, which for the most part characterize active chromatin domains. Lastly, genes that are stably induced or repressed (clusters 5-7 and 9-11) or transiently up- or down-regulated (clusters 12-19) show similar chromatin state enrichment level regardless of the time point at which gene expression changes are detected (Figure 3B, blue boxes 1 and 2). Thus, gene up- or down-regulation, and timing thereof, do not affect the global chromatin state load in the gene regions implicated.

Our results indicate that based on our HMM, chromatin signature complexity positively correlates with gene expression level, more so than with a switch in expression pattern such as activation or repression. This implies that timing of gene activation or repression is not determined by global epigenetic load; chromatin state enrichment is rather related to gene expression level.

### Temporal dynamics of chromatin states

**Figure 4:**
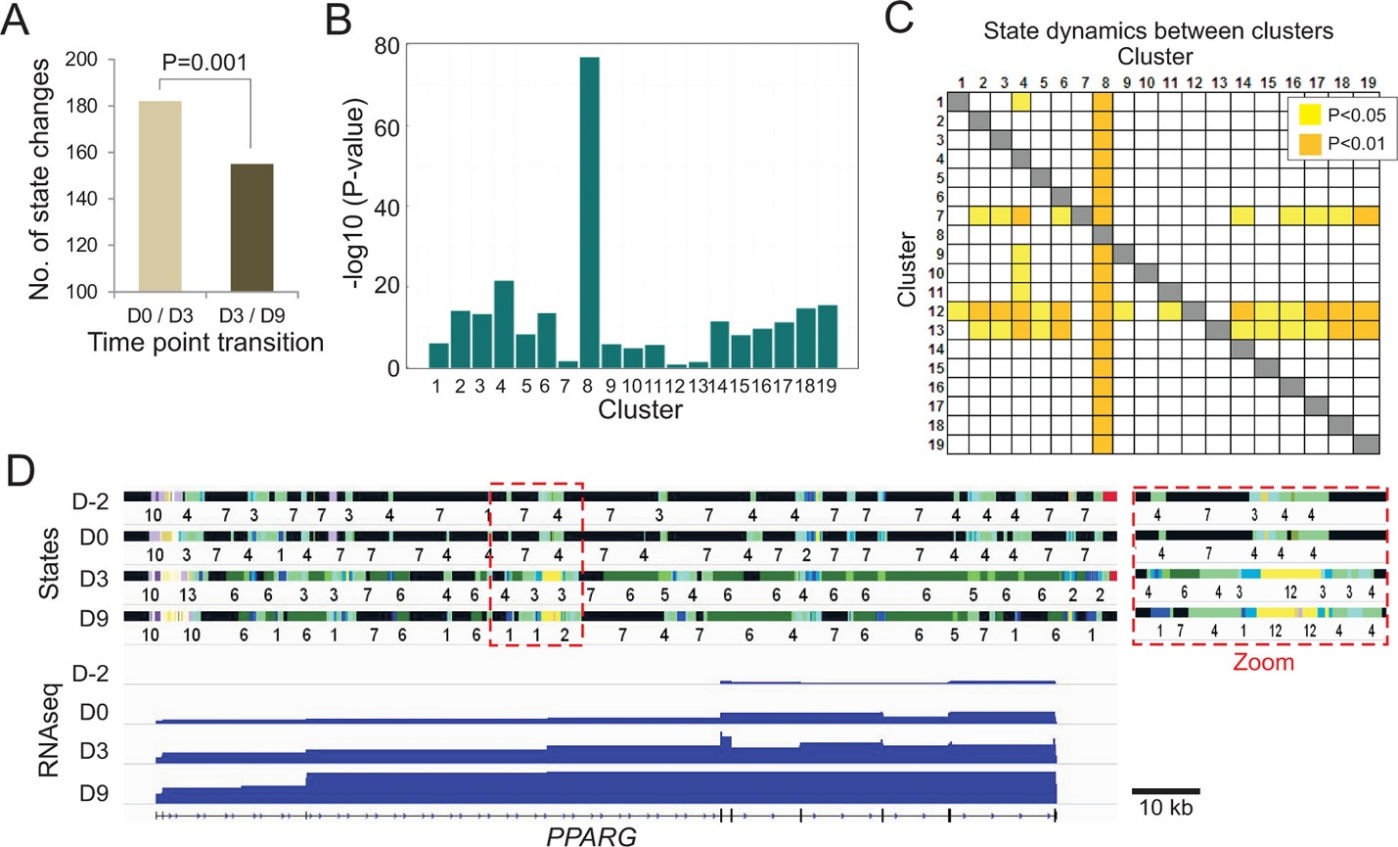
**Temporal and dynamic changes in chromatin states. (A)** Numbers of significant chromatin state changes (all clusters confounded) at the D0/D3 and D3/D9 transitions (t-test with Bonferroni correction). **(B)** Dynamics of chromatin states in each expression cluster. Graphs represent the sum of changes in P-values for each cluster. **(C)** Matrix of significant differences in chromatin state dynamics between clusters: clusters in columns (top) show greater state dynamics than clusters in rows (left). P-values for **(B)** and **(C)** are shown in Additional file 2: Table S6B (one-sided Wilcoxon tests). **(D)** Browser view of chromatin state changes and RNA-seq profiles at the *PPARG* locus during differentiation; the red boxed area is enlarged (zoom, right).

The temporal chromatin state maps we generated enable a dynamic analysis of chromatin state enrichment in the course of differentiation. We first find that the total number of significant state alterations between differentiation time points (Figure 3A, dots) is greater after adipogenic induction (D0-D3 transition) than during progression towards an adipogenic phenotype (D3-D9 transition; Figure 4A; P ≤ 0.001, t-test with Bonferroni correction). This suggests that once adipogenic commitment is initiated, patterns of chromatin modifications tend to stabilize.

Next, we compared global chromatin state dynamics between expression clusters by calculating the P-value of differences in state enrichment levels between time points in each cluster, averaging these P-values within clusters, and comparing cluster-averaged P-values. We first find that strongly expressed genes, in addition to being heavily epigenetically marked, display highly dynamic chromatin; in other words, states are locally dynamically changing between differentiation time points (Figure 3A, dots; Figure 4B, clusters 2-4). Second, chromatin states at genes that are expressed and further up-regulated during differentiation (cluster 8) are by far the most dynamic (Figure 4B,C; P = 2×10^−3^ to 4×10^−8^, one-sided Wilcoxon test; Additional file 2: Table S6B). For instance at the *PPARG* locus two dominant states, blank cs7 and weak enhancer state cs4 (H3K4me1) are substituted by active enhancer states (cs1, 2, 3, 12) and logically a state annotating transcribed gene bodies (cs6; H3K36me3; Figure 4D). Third, short temporal gene activation events, detected at one time point only, are associated with the lowest state dynamics (Figure 4B, clusters 12, 13). It is notably lower than the chromatin dynamics of genes showing longer lasting changes or several up- and down-regulation events changes (Figure 4B,C; clusters 5, 6, 14, 15 and 19). These observations suggest that chromatin is less prone to remodeling when genes are only transiently induced than when a more durable change occurs; this is again consistent with transcription being associated with dynamic chromatin states.

### Bivalent promoter states are the most dynamic during differentiation, while weak enhancers retain their chromatin signature

**Figure 5:**
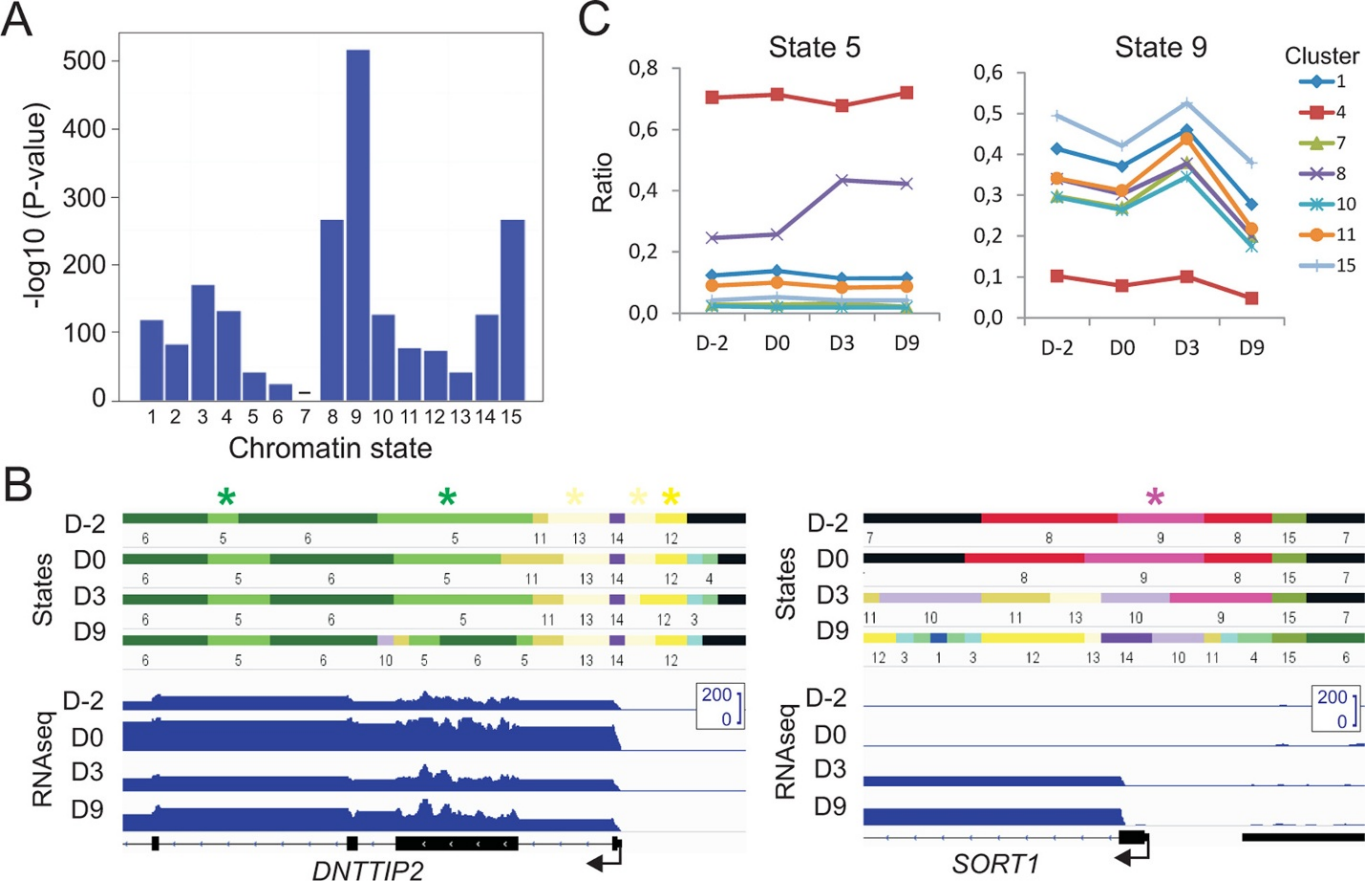
**Bivalent promoter states are the most dynamic during differentiation. (A)** Significance level of chromatin state change in all clusters during differentiation. **(B)** Browser views of chromatin state stability (cs5, 12, 13; top panel, stars) and dynamics (cs9, bottom panel, stars) throughout differentiation. Note the acquisition of cs9 (H3K4me2/H3K27me3) on the *SORT1* promoter on D0. RNA-seq profiles are also shown (scale: 0-200 FKPM). Window sizes shown: top, 8 kb; bottom, 6 kb. **(C)** Chromatin state enrichment profiles during adipogenic differentiation, illustrating the low and high dynamics of cs5 (left) and cs9 (right), respectively, for genes in the various clusters shown.

Analysis of significant temporal changes in individual chromatin states during differentiation reveals the dynamic nature of specific promoter states while in contrast, enhancer states are more stable. We find that, together with cs6 (H3K36me3), cs5 (H3K4me1/H3K36me3) is the least dynamic: it shows the lowest frequency of change throughout clusters (see Figure 3A) and changes of least significance between time points (Figure 5A). State 5 is annotated as ‘enhancer in active gene bodies’ (see Figure 2A), and reflects the variable localization of enhancers relative to the promoters they regulate [3]. Stability of cs5 during adipogenesis is exemplified by the steadily expressed *DNTTIP2* locus (Figure 5B) and is evidenced for several gene expression modules (Figure 5C). Additional enhancer states marked by H3K4me1, such as cs1, cs2 and cs4 (a ‘weak’ enhancer state) [21], or cs12 and cs13, are also among the temporally least dynamic (Figure 5A,B). State 1, an active enhancer state marked by H3K4me1 and H3K27ac, is more dynamic than weak enhancer states (Figure 5A; P = 0.049, two-sample Wilcoxon test): this may be explained by the connection between H3K27ac and enhancer activity, i.e. expression of the gene(s) it regulates [31]. We infer from these data that enhancers retain epigenetic identity through H3K4me1 marking in the adipogenic lineage.

In contrast to enhancer states, the ‘bivalent’ promoter state cs9 (H3K4me2/H3K27me3) is the most dynamic during differentiation (Figure 5A; P = 0.02 to 2.6x10^−6^). This is exemplified by the *SORT1* promoter (Figure 5B), controlling developmental expression of sortilin-1, involved in lipoprotein metabolism [32]. The dynamics of cs9 is consistent with enrichment of this apparently bivalent state on developmentally-regulated promoters in embryonic stem cells [33, 34] and in ASCs (data not shown), and its resolution into a H3K4 or K27 methylated state coincident with promoter activation or repression, respectively.

The temporal relationship between cs9 and transcript levels during differentiation is complex (Figure 5C) and reflects both a regulatory and priming role of this state on developmental gene expression. For instance, increase in cs9 from D0 to D3 on genes activated on D9, when cs9 load declines (cluster 7; Figures 2A and 5A), suggests that bivalent promoters are marked early during differentiation, prior to gene activation. In contrast, cs9 may mark expressed genes for repression (Figure 5C, cluster 11) or, consistent with a recent report [35], it may target genes after transcriptional inactivation is initiated (Figure 5C, cluster 10). cs9 load can also parallel transcript levels during differentiation (e.g. cluster 15; Figure 5C). Our data suggest that temporal dynamics of cs9 is linked to the H3K27me3 mark, as cs11, 12 and 13, promoter states deprived of H3K27me3, are less dynamic than cs9 (Figure 5A; P = 2.6×10^−5^ to 3.9×10^−6^). The temporal dynamics of the bivalent promoter state cs9 identified here infers its sensitivity to fluctuating changes in response to environmental stimuli.

## Discussion

We report a 15-chromatin state HMM which describes temporal changes in chromatin signatures in relation to gene expression patterns throughout adipogenic differentiation of human primary ASCs. Identification of coherent gene expression modules and of chromatin states fitting these modules demonstrates the complexity and dynamic nature of hPTM combinations during adipogenesis. These expression modules reveal distinct patterns including sustained expression, up- and down-regulation transitions, single-pulse patterns and oscillatory patterns. Interestingly, single-pulse expression patterns prevail in responses to stimuli [36], suggesting an adaptive response of ASCs to environmental changes such as cell cycle exit and adipogenic induction. These pulses initiate a downstream cascade of expression changes with sequential offsets which may involve feed-forward loops [37].

**Figure 6:**
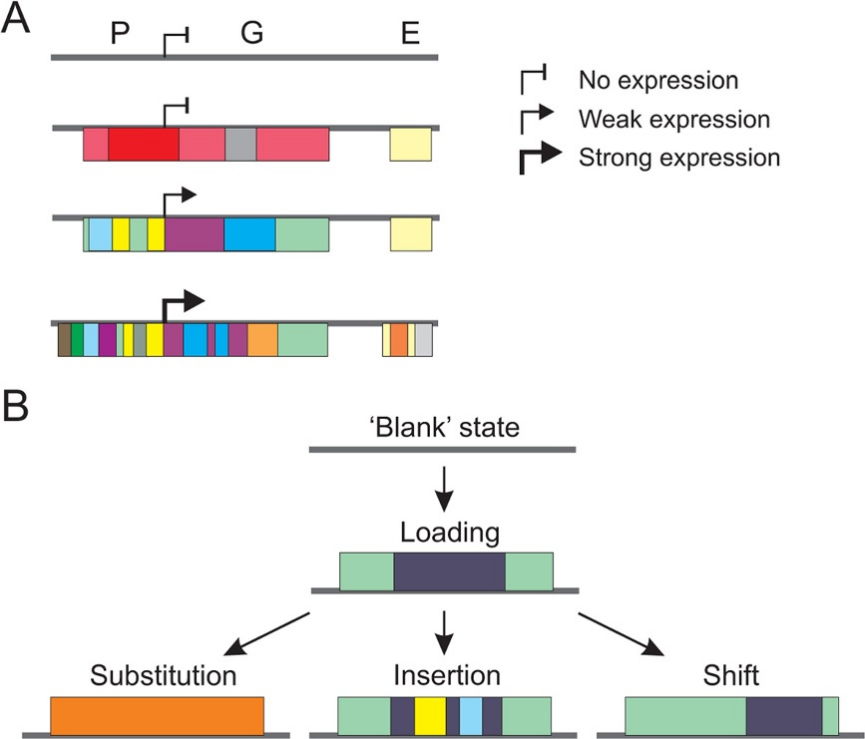
**A model of temporal patterns of chromatin state changes during differentiation. (A)** Increased gene expression level is associated with enhanced enrichment in chromatin states (color bars). Promoter regions (P) are particularly prone to modifications. G, gene body; E, enhancer. **(B)** Patterns of chromatin state dynamics during adipogenic differentiation. A blank state is commonly temporally followed by loading of chromatin states (color bars). States can then be replaced by another (substitution), become a site of emergence of one or more other states (insertion), or may shift in position (shift).

Active chromatin regions display tremendous diversity in epigenetic signatures (Figure 6A), suggestive of plasticity and amenability to remodeling. This complexity would be anticipated from the modifications examined, which mostly mark active chromatin. Our findings nonetheless also show the remarkable dynamics of these states at a given locus in the cell populations analyzed. Clearly, the diversity of chromatin state patterns may reflect complexity in a population of cells inasmuch as complexity within each cell in the population. Support to this interpretation is lent by a recent single-cell RNA-seq study of cell differentiation [29]. Single-cell quantitative epigenetic analyses would help clarify this issue but remain currently inaccessible at the level of resolution required. Gene expression may regardless play an important role in generating this chromatin diversity, perhaps by providing a chromatin template accessible for histone modifiers. The functional interplay between chromatin modifiers, hPTMs and transcriptional outputs has been difficult to underpin because this relationship is development- and locus-dependent [3, 10, 38, 39]). Chromatin engineering strategies may prove useful in dissecting the effect of histone modifications on gene expression [40].

Gene activation and inactivation is also associated with a remodeling of hPTMs, though not always concomitant to expression changes. Both the extent of chromatin modifications and chromatin state dynamics are lower in gene regions that are transiently up- or down-regulated than in highly active domains. This suggests that temporal gene expression is under control of factors other than hPTMs in a pre-disposed chromatin environment [16]. Supporting this idea, ‘stand-by’ occupancy of regulatory regions by pioneer transcription factors precedes activation of developmentally-controlled genes, conferring transcriptional competence [41–43]. Cooperative binding of transcription factors also plays a key role in the induction of adipogenic genes [15, 44]. Further, the correlation between transcription factor binding and target gene activation timing [45], together with differences in transcription factor affinity [46], could underlie the gene activation offsets within the adipogenic program.

Our temporal HMM reveals several patterns of chromatin state changes during adipogenic differentiation (Figure 6B). (i) The simplest is the formation of one or more state from a blank state, which is often detected at loci that become activated. (ii) Conversely, upon or following gene inactivation, one or more state can be lost, giving rise to a ‘blank’ state. (iii) One state can also be substituted by another: this occurs in the form of replacement of one state by another or by insertion of a state into a larger pre-existing domain. This is illustrated by the introduction of enhancer sites within gene bodies concomitant with gene up-regulation, such as our observations on *PPARG*. (iv) States may also spatially shift over a limited area, leading to the perception of local replacement; this may represent a regulated mechanism or a stochastic event of functional importance for proper gene regulation [47]. Restricted positional shifts of chromatin states are consistent with nucleosome sliding and histone replacement (along with their PTMs) events that occur on promoters and bodies of active genes [48–51].

The finding that the bivalent promoter state cs9 is the most dynamic during adipogenesis is surprising because promoter states tend to be conserved between cell types [21]. cs9 dynamics seems to be linked to the Polycomb-associated H3K27me3 mark, as promoter states with no H3K27 methylation are significantly less prone to change. The dynamic nature of Polycomb repressor complex 2 and of ensuing H3K27 trimethylation has been documented [52, 53]. Notably, an important caveat in the identification of a ‘bivalent’ state is that it remains unclear whether co-methylation on K4 and K27 occurs at the same locus or whether this state reflects heterogeneity in the cell populations. HMM data are compatible with both interpretations because input data into the model result from epigenomic information from not only all cells in a given population but also from all cell populations (i.e. here, all time points) examined [19]. Gain of cs9 on developmentally-regulated genes before transcriptional activation, or conversely loss of cs9 (i.e. loss of K4 or K27 methylation) reinforces the importance of Polycomb-mediated marking for proper adipogenic differentiation [54].

Inasmuch as promoter bivalency may precede developmental gene activation, *de novo* enhancer establishment by H3K4me1 marking emerges as a predictor of lineage-specific enhancer usage and downstream expression of the gene(s) it regulates [3, 10]. Thousands of H3K4me1-marked enhancers found in undifferentiated ASCs are retained through differentiation, in line with the adipogenic commitment of ASCs [55, 56]. In contrast, active enhancers (H3K4me1/H3K27ac; cs1 and cs2) are more dynamic, in keeping with the cell type specificity of H3K27ac [3, 10, 23]. A functional advantage in maintaining enhancer identity within a lineage may be by favoring the formation of ‘hubs’ of signal integration and relay to efficiently remodel chromatin and activate lineage-specific gene networks [17, 57, 58].

A remaining question is the origin of the multiple histone modifications detected on promoters and enhancers, including some known to commonly mark either element. This may reflect the embedding of enhancers in distinct domains such as promoters or transcribed exons, illustrating the variable localization of enhancer elements relative to promoters and genes they modulate [3]. Recent evidence indicates that enhancers interact with other elements, particularly promoters, through 3-dimensional chromatin looping [59–62]. Future studies will be important to determine the extent to which three-dimensional conformation of the genome impacts chromatin states, their developmental transitions and gene expression.

## Conclusions

Genome-scale modeling of chromatin into 15 states in the course of adipogenic differentiation demonstrates a hyper-dynamic nature of bivalent promoter states, which underlies distinct and coherent gene expression cohorts. Our results take our understanding of transitions in chromatin organization through the genome and in a developmental context to a new level, and constitute a resource enabling the assessment of possibilities to manipulate the adipogenic differentiation program.

## Methods

### Cells and adipogenic induction

Human adipose tissue stromal cells ASCs [63] were cultured under proliferative conditions in DMEM/F12 (Life Technologies) containing 10% FBS, 20 ng/ml basic fibroblast growth factor and 10 ng/ml epidermal growth factor (Sigma-Aldrich). Cells at passage 6-7 were used for differentiation in two independent experiments. Two days prior to adipogenic induction, cells were harvested and either used for analysis (D-2 time point), or reseeded to confluency in DMEM/F12/10% FBS without growth factors. Adipogenesis was induced on D0 by adding 0.5 μM 1-methyl-3 isobutylxanthine (Dumex Alpharma), 1 μM dexamethasone (Dumex Alpharma), 10 μg/ml insulin (Sigma-Aldrich) and 200 μM indomethacin (Dumex Alpharma) for up to 9 days [63].

### RNA isolation and RNA-sequencing

Total RNA was isolated on D-2, D0, D3 and D9 of adipogenic stimulation using the Ambion TRIzol® Reagent RNA extraction kit (Life Technologies). Libraries were prepared according to the Illumina protocol and sequenced to generate 100 base pair paired-end reads on an Illumina HiSeq2500. RNA-seq reads were processed using the Tuxedo pipeline [64]. TopHat [65] was used to align reads with no mismatch against human genome UCSC hg19 with default settings, applying the bowtie2 [66] preset ‘-very sensitive’. Cufflinks and cuffdiff were run using default settings and bias correction. Results were analyzed and visualized in R through cummeRbund. Genes with a log (fold change) > 2 and α < 0.05 were considered as differentially expressed. Coefficient of variation (CV^2^) was plotted against log (FPKM) (fragments per kilobase of exon per million fragments mapped) to identify differences in distribution of expression patterns. Principal component analysis was done using the first two principal components. Additional scripting was done in Perl or R.

### Clustering genes based on expression patterns

Genes used for clustering were obtained from the Tuxedo protocol with FPKM > 0 at at least one time point examined. Genes with similar expression patterns were clustered through hierarchical clustering using hclust in R. Gene Ontology enrichment analysis was done in Gorilla using default parameters [67].

### Attributing chromatin states to genes and gene expression clusters

ChIP-seq datasets of hPTMs and CTCF were obtained from a previous study [18] using a similar cell source, differentiation protocol and time line. ChIP-seq reads were re-mapped using bowtie and ‘-best’ default settings. hPTM and CTCF enrichment data were converted into chromatin states in consecutive 200-base pair bins using ChromHMM [19]. Options were selected to learn a 15-state model using the Baum-Welch training algorithm. States were linked to genes by computing the presence of a given state in the gene body plus a 10 kb extension upstream and downstream (‘gene ± 10 kb’) to take into account regulatory regions. This 10 kb value was set to 50% of the upper quantile of the distance of a changing chromatin state to the nearest gene/upstream or downstream) during differentiation. We found the mean distance to be 20 kb, calculated from the following data: 22.31 kb at the D-2/D0 transition, 22.52 kb at the D0/D3 transition, and 22.81 kb at the D3/D9 transition (data not shown). Note that increasing the extension window size from 10 kb to 22 kb did not significantly alter the number of chromatin states attributed to genes (data not shown). Chromatin state counts were normalized between gene expression clusters by dividing the number of genes (±10 kb) containing a given chromatin state at each differentiation time point by the total number of genes in each entire expression cluster. This generated ‘gene ratios’, which were used in statistical analyses. Normalization enabled a comparison of chromatin state levels within and between gene expression clusters.

### Statistical analyses

Statistical analyses were done in R. Regression and correlation analysis of chromatin state enrichment as a function of gene expression level was done using the Pearson method from numbers of chromatin states in gene regions (gene ± 10 kb; see above) and log (FPKM + 1) values. P-values for differences in enrichment of a chromatin state between differentiation time points were calculated using parametric one sample t-tests and Bonferroni correction to remove false positives, comparing each gene ratio difference to the overall difference in ratios in the cluster. Differences between clusters for chromatin state enrichment were identified using a non-parametric two sample Wilcoxon test and Bonferroni correction to remove false positives, as samples showed a skewed distribution; to this end, we used gene ratios at each time point for one cluster and compared them to all other clusters in a pair-wise manner. Differences in overall significance of chromatin state changes between clusters were tested by a non-parametric one-sided Wilcoxon test since distribution of the P-values was skewed. Graphs identifying the overall differences in the significance of P-values (chromatin state changes) were generated by computing the -log10 value of the sum of the P-values.

### Data viewing

Browser views of gene tracks, ChIP-seq data and chromatin states are shown using Integrated Genomics Viewer (IGV; broadinstitute.org/igv) [68]. Unless otherwise stated genes considered in the analyses are from the Illumina iGenomes gene annotation with UCSC data source for hg19 (support.illumina.com/sequencing/sequencing_software/igenome.ilmn).

### Data access

Our RNA-seq data are available from NCBI GEO accession number GSE60237. Published ChIP-seq data [18] were from NCBI GEO accession number GSE20752.

## Electronic supplementary material

Additional file 1: **A PDF file containing two figures including. Figure S1.** Transcriptional changes elicited by adipogenic differentiation of human ASCs segregate undifferentiated cells from adipogenic stimulated cells; **Figure S2.** Genome coverage of chromatin states identified and relation to gene expression. **Table S1.** Numbers of RNA-seq reads mapped. (PDF 361 KB)

Additional file 2: **A zip file containing five Excel tables. Table S2.** Genes up- or down-regulated at each stage of differentiation; **Table S3.** GO terms enriched among genes up- or down-regulated at each stage of differentiation; **Table S4.** GO terms enriched among genes included in the 19 expression clusters; **Table S5**: Genes included in the 19 expression clusters; **Table S6**: Comparisons of enrichment in chromatin states and of chromatin state dynamics between expression clusters. Tables show P-values of (A) comparisons of enrichment in chromatin states (gene ratios; Wilcoxon test with Bonferroni correction and (B) comparisons of chromatin state dynamics (gene ratio differences; one-sided Wilcoxon tests). (ZIP 1 MB)

## Abbreviations

ASC: Adipose tissue stromal cell
ChIP: Chromatin immunoprecipitation
ChIP-seq: ChIP and high-throughput sequencing
cs: chromatin state
RNA-seq: RNA-sequencing
TSS: Transcription start site.
