# Supplementary material for: A hyper-dynamic nature of bivalent promoter states underlies coordinated developmental gene expression modules
Source: BMC Genomics. 2014 Dec 30;15(1):1186. doi: 10.1186/1471-2164-15-1186 (PMC4320513; doi:10.1186/1471-2164-15-1186)
Supplement: Supplementary file 1 — Additional file 1: A PDF file containing two figures including. Figure S1. Transcriptional changes elicited by adipogenic differentiation of human ASCs segregate undifferentiated cells from adipogenic stimulated cells; Figure S2. Genome coverage of chromatin states identified and relation to gene expression. Table S1. Numbers of RNA-seq reads mapped. (PDF 361 KB) [file 12864_2014_6925_MOESM1_ESM.pdf]

A hyper-dynamic nature of bivalent promoter states underlies coordinated developmental gene expression modules

Akshay Shah, Anja Oldenburg and Philippe Collas

Integrated Chromatin, Autophagy and Metabolism (iCAM) Program, Institute of Basic Medical Sciences, Faculty of Medicine, University of Oslo, and Norwegian Center for Stem Cell Research, Oslo University Hospital, 0317 Oslo, Norway

Additional files

Additional file 1. A PDF file containing two figures and one table including: **Figure S1:** Transcriptional changes elicited by adipogenic differentiation of human ASCs segregate undifferentiated cells from adipogenic stimulated cells; **Figure S2:** Genome coverage of chromatin states identified and relation to gene expression level. **Table S1.** Numbers of RNA-seq reads mapped.

Additional file 2. A zip file containing a set of 5 Excel tables: **Table S2:** Genes up- or down-regulated at each stage of differentiation; **Table S3:** GO terms enriched among genes up- or down-regulated at each stage of differentiation; **Table S4:** GO terms enriched among genes included in the 19 expression clusters; **Table S5:** Genes included in the 19 expression clusters; **Table S6:** Comparisons of enrichment in chromatin states and of chromatin state dynamics between gene expression clusters. Tables show P-values of **(A)** comparisons of enrichment in chromatin states (gene ratios; Wilcoxon test with Bonferroni correction and **(B)** comparisons of chromatin state dynamics (gene ratio differences; one-sided Wilcoxon tests).

Additional file 1

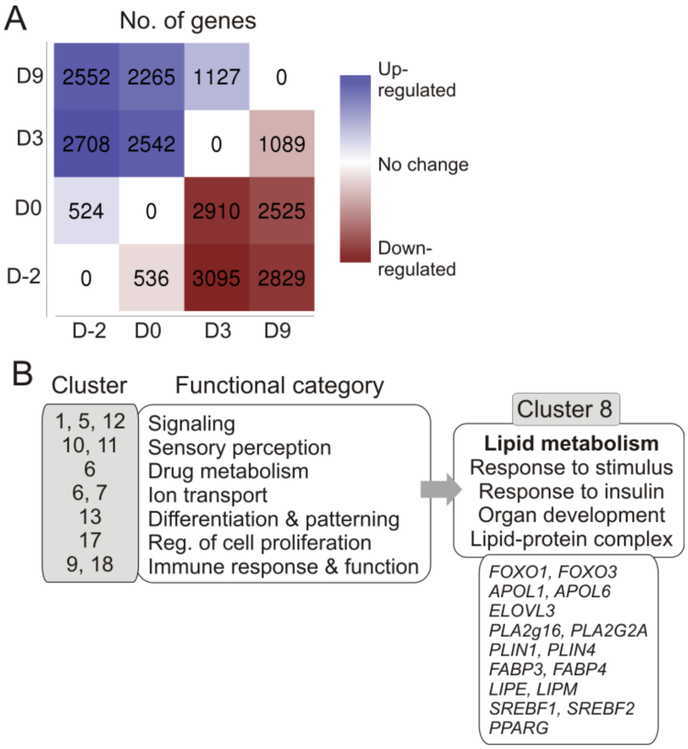

Figure S1 Transcriptional changes elicited by adipogenic differentiation segregate undifferentiated cells from adipogenic stimulated cells. **(A)** Numbers of differentially up- and down-regulated genes (fold change > 2, $\alpha < 0.05$) at each differentiation time point. **(B)** Main functional GO categories associated with indicated gene expression clusters. Cluster 8 contains 265 genes including many genes required for adipogenic induction and lipid processing such as FOXO transcription factors (*FOXO1*, *FOXO3*), apolipoproteins (*APOL3*, *APOL6*), fatty acid elongase 3 (*ELOVL3*), phospholipases (*PLA2G16*, *PLA2G2A*), perilipins (*PLIN1*, *PLIN4*), fatty acid binding proteins (*FABP3*, *FABP4*), hormone-sensitive lipases (*LIPE*, *LIPM*), *PPARG*, sterol regulatory element binding transcription factors 1 and 2 (*SREBF1*, *SREBF2*), and transporters (see Supplemental Table S4 for gene lists).

Additional file 1

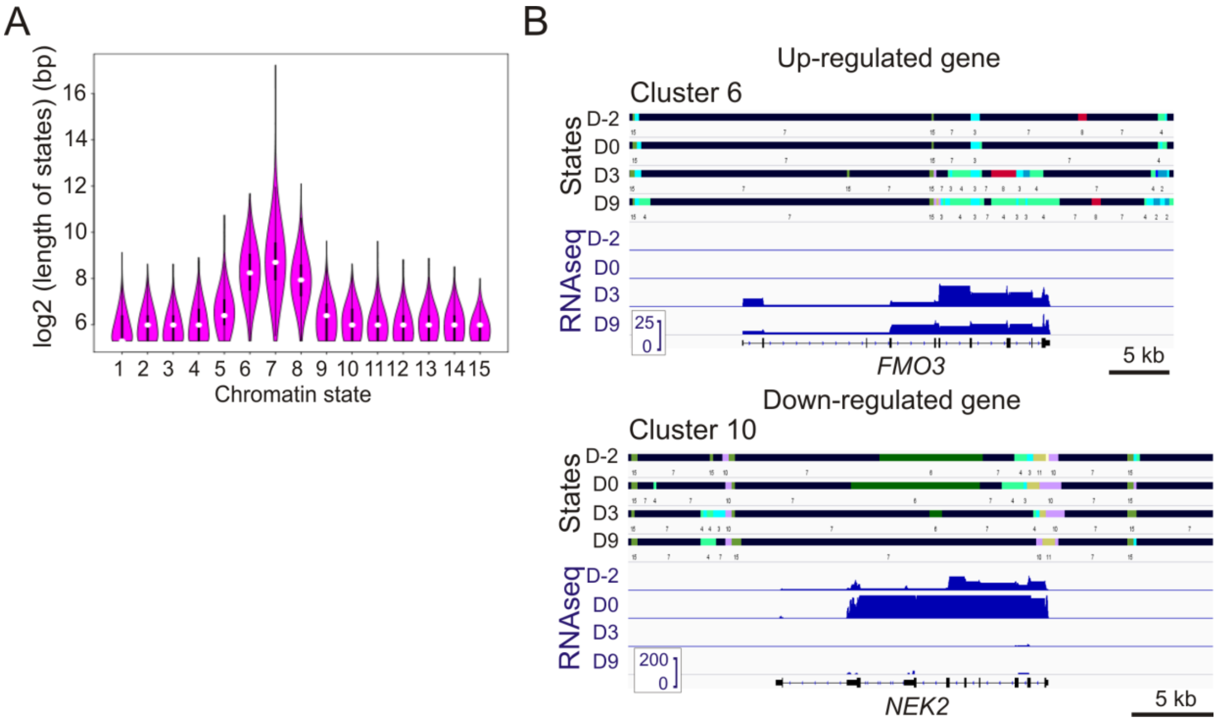

Figure S2 Genome coverage of chromatin states and relation to gene expression. **(A)** Median and distribution of length of chromatin states learned in our model. **(B)** Brower views of chromatin states and RNA-seq expression levels for an up-regulated gene of cluster 6 and a down-regulated gene of cluster 10. FPKM ranges are shown.

Additional file 1

Supplemental Table S1. Numbers of RNA-seq reads mapped

Differentiation time point	No. reads	No. paired alignments mapped
D-2	40 702 891	30 478 008
D0	102 000 288	95 610 686
D3	93 577 306	72 047 076
D9	72 755 478	58 610 274
